# The impact of COVID‐19 on the safety, housing stability, and mental health of unstably housed domestic violence survivors

**DOI:** 10.1002/jcop.22765

**Published:** 2021-12-18

**Authors:** Danielle Chiaramonte, Cortney Simmons, Noora Hamdan, Oyesola Oluwafunmilayo Ayeni, Gabriela López‐Zerón, Adam Farero, Mackenzie Sprecher, Cris M. Sullivan

**Affiliations:** ^1^ Department of Psychiatry, Department of Psychology Yale University New Haven Connecticut USA; ^2^ Department of Pyschology Drakeford, Scott, & Associates, LLC Upper Marlboro Maryland USA; ^3^ Department of Psychology University of Michigan Ann Arbor Michigan USA

**Keywords:** COVID‐19, intimate partner violence, longitudinal

## Abstract

Using data from an ongoing longitudinal study, we examined the impact of the COVID‐19 stay‐at‐home orders on a racially diverse population of unstably housed domestic violence (DV) survivors over time. Specifically, we examined survivors' safety, housing stability, and mental health before, during, and after the onset of COVID‐19, and how demographic, social, and familial factors attenuated or exacerbated the effect of the stay‐at‐home orders. Approximately 300 participants were initially interviewed after they sought services from a DV agency, and then again, every 6 months over 2 years. COVID‐19 stay‐at‐home orders occurred midway through the completion of this multi‐year study. Longitudinal mixed effects models were estimated to examine the impact of COVID‐19 on the safety, housing stability, and mental health of survivors over time. We also examined models with several time‐varying (e.g., employment, income, social support, and number of children) and time‐invariant (baseline outcome scores, racial/ethnic identity, education, and disability status) control variables. Results revealed that safety, housing stability and mental health were improving for study participants before the onset of the COVID‐19 pandemic but plateaued after the stay‐at‐home orders were issued. Experiences of abuse, housing instability, and mental health symptomatology did not worsen as a result of the COVID‐19 stay‐at‐home orders. Notably, social support and housing services emerged as important predictors of outcomes, such that participants who received housing‐related services and greater social support reported less abuse, less housing instability, and lower mental health distress. COVID‐19 temporarily disrupted the positive trajectory unstably housed DV survivors were experiencing in regard to safety, housing stability and mental health. These findings provide critical insight into the importance of service access during and after global catastrophes. Additional resources and support may be helpful in assisting survivors to return to their pre‐pandemic recovery and growth trajectories.

## 1 INTRODUCTION

The COVID‐19 pandemic has demonstrably impacted lives of individuals and families across the world. Scores of people lost their jobs (Béland et al., 2020; Cho & Winters, 2020), social support networks (Marroquín et al., 2020), and many community‐based organizations limited their services (Wood et al., 2021). This global health emergency has triggered widespread uncertainty for the future and economic repercussions that have negatively affected individual's mental health (Bhattacharjee & Acharya, 2020; Sediri et al., 2020) and housing stability (Buckle et al., 2020; Jones & Grigsby‐Toussaint, 2020). For those already experiencing higher oppression and discrimination, the pandemic has exacerbated living conditions, economic stability, and general wellbeing (Parker & Leviten‐Reid, 2021; Rauhaus et al., 2020). Such negative outcomes have been particularly detrimental to those who had already faced housing instability before the onset of the pandemic (Khan et al., 2020; Tsai et al., 2021).

Among those significantly impacted by COVID‐19 have been domestic violence (DV) survivors. During the early stages of the pandemic, evidence emerged of the alarming increase in DV as a result of the initial stay‐at‐home orders across the globe (Boserup et al., 2020; Bracewell et al., 2020; Leslie & Wilson, 2020; Nix & Richards, 2021; Piquero et al., 2020). Many DV survivors already faced significant mental health and housing challenges, so the compounding impact of the pandemic resulted in additional instability (Beydoun et al., 2012; Spencer et al., 2019; Trevillion et al., 2012). Evidence suggests that the financial distress and isolation brought about by the pandemic increased abuse or made it more difficult to leave. One study examining online posts found that several perpetrators used the COVID‐19 pandemic as an additional tool for abuse (Lyons & Brewer, 2021). Further, social isolation seems to have been particularly challenging to navigate for DV survivors who may have experienced disruptions to formal social supports such as therapists and DV services, as well as informal supports from friends and family (Bradley et al., 2020; Lyons & Brewer, 2021; Mojahed et al., 2021; van Gelder et al., 2020). Given what we know about the pandemic's impact on economic stability and mental health in general, there is also concern that these factors may have been exacerbated for DV survivors who were already precariously housed before the pandemic

The present study follows a particular segment of DV survivors over the course of the pandemic: those who have sought services for DV and experienced housing instability. To date, no other study has considered the impact of the COVID‐19 pandemic on this particular population of help‐seeking, unstably housed DV survivors. Yet, this examination is critical given that intimate partner violence is the leading cause of homelessness (Pavao et al., 2007). In addition, the present study sheds light on the impact of a global health emergency on an ongoing, housing‐focused DV intervention.

### 1.1 Current study

The purpose of this study was to examine the impact of COVID‐19 on the safety, housing stability, and mental health of a racially diverse population of unstably housed DV survivors over 24 months. While a small number of other longitudinal studies have compared their entire samples at timepoints pre‐ and postpandemic (e.g., Nearchou et al., 2020; Niedzwiedz et al., 2021) this is the first study to our knowledge where COVID‐19 occurred midway through the completion of a multi‐year study, with some participants having completed a timepoint before the pandemic and others completing the same timepoint afterward. For example, 42% of the sample completed their 18‐month interview before the pandemic and 58% completed it afterward.

Given the specificity with which we measured the onset of the COVID‐19 pandemic for each participant, and the fact that the pandemic began midway through longitudinal data collection, we are in the unique position to parse out the influence of COVID‐19 versus other factors impacting DV survivors' well‐being. Specifically, we hypothesized that the pandemic would have a negative impact on survivor safety, housing stability, and mental health (depression, anxiety, and posttraumatic stress disorder [PTSD]) over time. We also expected that demographic, social, and familial factors would attenuate or exacerbate the effect of the stay‐at‐home orders.

## 2 METHODS

Data for these analyses are from a larger, ongoing longitudinal study examining the impacts of services on safety, housing stability, and well‐being for homeless and unstably housed DV survivors.

### 2.1 Participants and procedures

Participants were 97% cisgender female and predominantly of minority racial and ethnic groups (64%). Most of the participants identified as heterosexual (86%). Seventy‐three percent had a prior history of homelessness, and the average cumulative amount of time participants had spent homeless before entering the study was just over 2 years. At baseline, 42% of the sample were homeless (i.e., living in a shelter or unsheltered homeless) while the other 58% of participants were in highly precarious housing situations. More detailed descriptive information about the sample is located in Table 1.

**Table 1:** Descriptive statistics for covariates and outcome variables

	Baseline		6 Months		12 Months		18 Months		24 Months	
	N/M	%/*SD*	N/M	%/*SD*	N/M	%/*SD*	N/M	%/*SD*	N/M	%/*SD*
**Time invariant covariates**										
Minoritized racial or ethnic identity	261	64	–	–	–	–	–	–	–	–
Living with disability or disabling condition	143	35	–	–	–	–	–	–	–	–
Education			–	–	–	–	–	–	–	–
Less than high school degree	117	28.82	–	–	–	–	–	–	–	–
GED, high school graduate	89	21.92	–	–	–	–	–	–	–	–
Some college, associate's, vocational, certificate	147	36.21	–	–	–	–	–	–	–	–
Higher education	53	13.05	–	–	–	–	–	–	–	–
Housing‐focused DV services			–	–	–	–	–	–	–	–
Received	224	60.5	–	–	–	–	–	–	–	–
Did not receive	159	41.5	–	–	–	–	–	–	–	–
English reading proficiency	2.66	0.73	–	–	–	–	–	–	–	–
**Time varying covariates**										
Employment										
Not employed	262	58.5	191	51.2	188	51.4	177	49.4	161	53.3
Parttime/seasonally employed	63	15.5	72	19.3	54	14.75	55	15.4	40	13.3
Employed fulltime	80	19.8	110	29.5	124	33.9	126	35.2	101	33.4
Number of children	1.48	1.32	1.40	1.39	1.43	1.36	1.43	1.35	1.35	1.34
Social support	3.28	1.15	3.48	1.18	3.52	1.12	3.57	1.14	3.50	1.16
Household Income	3.30	2.35	4.47	2.41	4.51	2.58	4.87	2.67	4.87	2.76
**Outcomes**										
**Safety**										
Physical abuse	1.29	1.09	0.29	0.60	0.18	0.51	0.15	0.47	0.10	0.29
Emotional abuse	2.07	1.31	0.60	0.92	0.51	0.81	0.37	0.70	0.44	0.78
Sexual abuse	1.16	1.51	0.18	0.65	0.14	0.56	0.12	0.56	0.09	0.37
Economic abuse	1.46	1.05	0.49	0.84	0.63	0.83	0.29	0.63	0.2	0.49
Stalking	2.25	1.60	1.11	1.34	0.84	1.18	0.70	1.09	0.52	0.92
**Housing instability**	4.74	1.66	3.52	2.05	2.53	2.12	2.25	1.99	1.79	1.96
**Mental health**										
Depression	12.99	6.73	10.17	6.61	9.25	6.70	8.60	6.48	8.55	6.81
Anxiety	12.16	6.28	9.54	6.19	8.82	6.30	8.32	6.16	8.34	6.50
PTSD	6.88	2.48	5.85	3.07	5.46	3.11	5.12	3.20	4.91	3.34

Abbreviations: DV, domestic violence; PTSD, posttraumatic stress disorder.

Participants were recruited into the study shortly after they had sought services from one of five DV agencies in the Pacific Northwest. Study eligibility included being a recent adult survivor of DV and being either homeless or unstably housed. Interviews were conducted in English or Spanish, depending on participant preference. Agency staff referred 438 eligible survivors to hear more about the study, and 406 (93%) agreed to participate. Participants were interviewed every 6 months over the course of 24 months (baseline, 6, 12, 18, 24 months) by highly trained interviewers, and were paid $50 per interview. Procedures were approved by the last author's institutional review board. The retention rate across time remained high (92% at 6 months, 91% at 12 months, 88% at 18 months and 89% at 24 months).

All participants had completed their baseline and 6‐month interviews before COVID‐19 was declared a worldwide pandemic (using March 15, 2020, as the start date when stay‐at‐home orders began), and one‐third of the sample had completed all five interviews across the 24 months. For the remainder of the sample, 20% completed their 12‐month interview after the pandemic began, 42% completed their 18‐month interview after the pandemic, and 67% completed the 24‐month interview after the pandemic began.

#### 2.1.1 Analytic sample

Data collection for the final 24‐month interview was still ongoing at the time of this analysis. Our final sample sizes at each timepoint were baseline: 406; 6‐month follow‐up interview: 375; 12‐month follow‐up interview: 369; 18‐month follow‐up interview: 359; 24‐month follow‐up interview: 306. Additionally, as the baseline interview was conducted shortly after participants reached out for services, the majority of participants had received services from a DV agency between the baseline and 6‐month timepoint. As a result, there were steep changes on safety, housing stability, and mental health between these two timepoints. Because the focus of this paper is on the impact of COVID‐19, and not the effectiveness of DV services, we removed the baseline scores from growth score trajectories to measure the impact of COVID‐19 more accurately within this population. Baseline scores on outcome variables were, however, added as covariates in the model.

#### 2.1.2 Covariates

In our analyses, we controlled for factors that could influence how the COVID‐19 pandemic impacted DV survivor safety, housing stability, and mental health. The pandemic impacted the lives of communities of Color more than White communities and low‐income households more than high‐income households (e.g., Artiga et al., 2020; Kantamneni, 2020). Given this, we controlled for identification with a minority race/ethnicity, and household income. We also controlled for highest educational attainment and ability to read English at baseline as both could impact survivors' safety and risk knowledge surrounding the pandemic (Hamaguchi et al., 2020). Next, we controlled for whether someone was employed at baseline, as those who were employed before the pandemic would have been eligible for unemployment assistance. Social support received throughout the pandemic was also controlled for, since those who were receiving such support might differ in their experience of the pandemic (i.e., stress, fear, etc.) from those without such support (Li et al., 2021). Additionally, we controlled for whether survivors had a disability, as those in the disabled community were at greater risk of more adverse symptoms if they were to contract COVID‐19 (Armitage & Nellums, 2020). We also controlled for whether survivors had children they were caring for over the course of the study, since the closing of schools and the necessity to plan childcare would not impact those without children (Adams et al., 2021). Last, we controlled for all baseline scores on our outcomes of interest since survivor trajectories could vary depending on their baseline positions.

### 2.2 Measures

In addition to demographics, the current analysis used the following measures to examine the unique impact of COVID‐19 on survivors' safety, housing stability, and mental health. Descriptive statistics for each of these variables is available in Table 1.

#### 2.2.1 Time

Participants were asked, during each interview, about events occurring since their prior interview. For those interviewed before March 15, 2020, COVID‐19 stay‐at‐home orders had not yet started. Those interviewed 6 months later (after September 15, 2020) would have been reflecting entirely on months impacted by the pandemic. For those interviewed between these dates, however, the time period on which they were reporting would contain one to five “pre‐pandemic” months and one to five “post‐pandemic” months. For example, someone who completed their 12‐month interview on March 31, 2020, would be reflecting back on five and a half “pre‐pandemic” months and only 2 weeks “post start of pandemic.” A participant completing their 12‐month interview on July 31, 2020, would be reflecting back on 6 weeks “pre‐pandemic” and four and a half months after the start of the pandemic. If, as hypothesized, length of time since the start of the pandemic is important to account for, we cannot consider these two individuals to have had a similar “dosage” of the pandemic. Therefore, the data wererestructured to account for the number of months before and after the onset of the COVID‐19 pandemic (see Table S1 in the Supporting Information Materials). For variables that had 6‐month recall periods (e.g., “In the last six months have you…”), data were restructured to 6‐month intervals before and after the onset of the pandemic. For outcomes with more immediate recall periods (e.g., “Over the last two weeks how often have you been bothered by…”), the data were restructured to 3‐month intervals after the COVID‐19 pandemic. By examining 3‐month intervals after the onset of the pandemic, we were able to observe more specific effects of the pandemic as time progressed.

#### 2.2.2 Outcome variables

##### Safety

During each interview, four common forms of DV (physical abuse, emotional abuse, stalking, and sexual abuse) were assessed using the 28‐item Composite Abuse Scale (CAS) (Hegarty et al., 1999; Loxton et al., 2013). Two CAS questions (“hang around outside your house” and “harass you at work”) were replaced with a new item (“repeatedly follow you, phone you, and/or show up at your house/work/other place”) to obtain indicators of stalking that were pertinent even if the participant was living with the abuser. Four additional items were added to the CAS to capture abuse not adequately measured in the original scale: (1) stalk you, (2) strangle you, (3) demand sex whether you wanted to or not, and (4) force sexual activity. Questions were asked within the format: “How often, if at all, did [abuser's name]: …” The original response options for the CAS, which ranged from “daily” to “never,” were modified to accommodate interviews occurring every 6 months: 0 = “never,” 1 = “once,” 2 = “several times or between 2 and 3× in the last 6 months,” 3 = “once a month,” 4 = “once a week,” and 5 = “daily.” The final measure included subscales measuring physical abuse (*α* = 0.91), emotional abuse (*α* = 0.91), sexual abuse (*α* = 0.92), and stalking (Cronbach's alpha = 0.84). Cronbach's alpha for the combined measure was 0.95.

The Revised Scale of Economic Abuse (SEA2; Adams et al., 2020) was used to measure tactics targeted toward endangering intimate partners' and ex‐partners' financial stability. Sample items included asking how often in the prior 6 months the abuser would “force or pressure you to give them your savings or other assets,” and “keep you from having a job or going to work.” Response options ranged from 0 = “never” to 4 = “quite often.” Cronbach's alpha for the measure was 0.91.

##### Housing instability

During each interview, housing instability was assessed using six items from the Housing Instability Index (Rollins et al., 2012). Items included “Do you expect that you will be able to stay in your current housing for the next 6 months?”, “How likely is it that you will be able to pay for your housing this month?”, “In the past 6 months, how many times have you moved?”, “In the past 6 months, have you had to live somewhere that you did not want to live?”, “ Have you had trouble getting housing in the past 6 months?”, “In the past 6 months, have you had difficulty (or were unable to) pay for your housing?” Additionally, participants were asked: “In the last 6 months, have you been homeless or had to live with family or friends to avoid being homeless?” Of the seven scale items, five were dichotomous (yes/no) and two were re‐coded to be dichotomous. For each item, 0 = more stable and 1 = less stable. Scores range from 0 to 7, with higher scores indicating greater instability. Cronbach's alpha for the index was 0.65.

##### Mental health

Depression was measured with the Patient Health Questionnaire (PHQ‐9; Kroenke et al., 2001). Participants were asked about the frequency of depressive symptoms (e.g., “feeling down, depressed, or hopeless”) over the previous 2 weeks (4‐point Likert scale 0 = not at all to 3 = nearly every day). Cronbach's alpha was 0.88.

Anxiety was determined with the Generalized Anxiety Disorder measure (GAD‐7; Spitzer et al., 2006). Items refer to the frequency of anxious feelings (e.g., “not being able to stop or control worrying”) over the previous 2 weeks (4‐point Likert scale 0 = not at all to 3 = nearly every day). Cronbach's alpha was 0.89.

Posttraumatic stress symptomatology was computed using the Trauma Screening Questionnaire (TSQ; Brewin et al., 2002). Items refer to physical or emotional responses to trauma (e.g., “upsetting thoughts about the event that have come into your mind against your will”) in the prior week. Response options were 0 (no) and 1 (yes), and a score of 6 or higher denotes the likelihood of experiencing PTSD. Cronbach's alpha was 0.76.

### 2.3 Covariates

#### 2.3.1 Time‐invariant covariates

##### Race/ethnicity

During the baseline interview, participants self‐identified their race and ethnicity. A dichotomous race/ethnicity variable was then generated for our models: 1 = one or more minority racial or ethnic identity; 0 = non‐Hispanic/Latinx White identity. The majority of the sample (*n* = 261) was categorized as one or more minority racial or ethnic identity.

##### Education

During the baseline interview, participants reported their highest educational achievement. A four‐category education variable was generated for analyses: 0 = Less than a high school degree; 1 = High school degree or GED; 2 = Some college, Associate's degree, vocational school/training certificate; and 3 = Bachelor's degree or higher.

##### Disability

During the baseline interview, participants reported if they had a physical disability or disabling condition (0 = No, 1 = Yes).

##### English reading proficiency

During the baseline interview, participants reported on their ability to read English using a 4‐point Likert scale (0 = “not at all”, 1 = “not well”, 2 = “okay”, and 3 = “very well”).

##### Household income

During the baseline interview, participants reported their annual household income from the following categories: (1) Under $5000, (2) $5000–$9999, (3) 10,000–$14,999, (4) $15,000–$24,999, (5) $25,000–$34,000, (6) $35,000–$49,999, (7) $50,000–$74,999, (8) $75,000–$99,999, (9) $100,000–$149,000, and (10) $150,000 or more.

##### Housing‐focused DV services

All the DV agencies offered a range of services to survivors (e.g., support groups, counseling, safety planning, advocacy), and two services were specific to increasing safe and stable housing (housing‐focused advocacy and flexible funding). Given resource constraints of agencies, participants may or may not have received these services, but given the likelihood of these services positively impacting safety and housing stability (Sullivan & Olsen, 2016; Thomas et al., 2020), we controlled for them in analyses. Just under a third of participants received both housing‐focused advocacy and flexible funding (30.7%), while 16.9% received housing‐focused advocacy but no funding, and 10.7% received funding but no housing‐focused advocacy. A third (33.3%) received other agency services and 8.3% received no services. A dichotomous variable was generated to control for the effects of these services: 0 = did not receive housing‐focused advocacy and/or funding, and 1 = received housing‐focused advocacy and/or funding.

#### 2.3.2 Time‐varying covariates

##### Employment

During each interview, participants were asked about their current employment status. Response options included: Employed, working 41 or more hours per week; Employed, working 30–40 h per week; Employed, working less than 30 h per week; Not employed, looking for work; Not employed, not looking for work; Retired; Disabled, not able to work; Employed seasonally. A three‐category employment variable was generated from the participants' responses: 0 = not employed (including disabled and retired); 1 = employed part‐time or seasonally; 2 = employed fulltime.

##### Number of minor children

During each interview, participants reported how many children they were caring for under the age of 18.

##### Social support

During each interview, social support was assessed using the 6‐item Medical Outcomes Study Social Support Survey (MOS‐SSS‐6; Holden et al., 2014). Prior research indicates the scale has high reliability in both English and Spanish (Gomez‐Campelo et al., 2014; Sherbourne & Stewart, 1991). The items consisted of questions regarding how confident the survivors feel about others in their lives who could support them in times of need (e.g., *How much of the time would you say you currently have someone in your life who could take you to the doctor*?). Response options ranged from:1 = “none of the time” to 5 = “all of the time.” Cronbach's alpha was 0.90.

### 2.4 Data analytic strategy

Longitudinal mixed effect models were estimated in Stata v17 to examine the effect of the COVID‐19 pandemic on safety, housing stability, and mental health symptomatology during the 2.5 years before and 1 year after the onset of the COVID‐19 pandemic. First, unconditional mixed effect models were estimated with time as the only predictor to examine time‐related changes in safety, housing stability, and mental health before and after the onset COVID‐19 pandemic. A random intercept was included for participant and time to allow for variation in change over time between and within participants. The unconditional models included time as a categorical predictor to examine differences in outcomes assessed at the onset of COVID‐19 pandemic and every other timepoint (Tables S2–S4). Second, conditional mixed effect models were estimated with time‐varying (e.g., employment, income, social support, and number of children) and time‐invariant (baseline outcome scores, racial/ethnic identity, education, and disability status) predictors. The reference timepoint in these models was the first timepoint of the pandemic (i.e., COVID‐19 onset).

## 3 RESULTS

### 3.1 Safety

**Figure 1:**
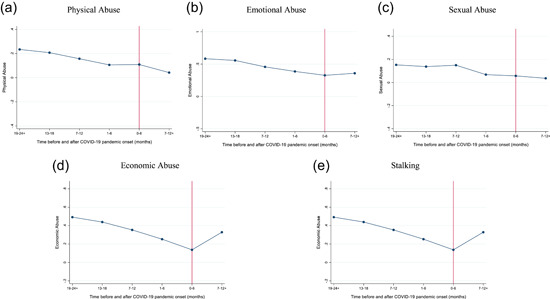
(a–e) Change in abuse over time: before and after the onset of COVID. Red line indicates the onset of COVID‐19

The unconditional mixed effect models indicated that all forms of abuse significantly decreased before the onset of the COVID‐19 (Table S2). After the onset of COVID‐19, there were no significant differences between physical abuse reported during the first three months of the pandemic and physical abuse reported during the subsequent timepoints (Table 2 and Figure 1a). Similar results were observed for emotional abuse, sexual abuse, economic abuse, and stalking, suggesting that COVID‐19 disrupted the downward trajectory of abuse (Table 2 and Figure 1b–e). The figures illustrate the changes in type of experiences of each type of abuse over time before and after the onset of COVID‐19.

**Table 2:** Conditional mixed effects models with covariates predicting abuse

	Physical abuse					Emotional abuse					Sexual abuse				
	*b*	*SE*	*p*	95% CI		*b*	*SE*	*p*	95% CI		*b*	*SE*	*p*	95% CI	
Time															
19–24+ months before COVID	0.13	0.04	0.01	0.04	0.21	0.26	0.07	0.00	0.11	0.40	0.10	0.05	0.06	0.00	0.19
13–18 months before COVID	0.10	0.04	0.01	0.02	0.18	0.23	0.07	0.00	0.10	0.36	0.08	0.05	0.08	0.01	0.17
7–12 months before COVID	0.05	0.04	0.21	−0.03	0.12	0.13	0.06	0.04	0.01	0.25	0.09	0.04	0.03	0.01	0.18
1–6 months before COVID	0.00	0.04	0.94	−0.08	0.07	0.06	0.06	0.35	−0.07	0.19	0.01	0.04	0.80	0.07	0.10
7–12+ months after COVID	−0.07	0.19	0.72	−0.43	0.30	0.03	0.31	0.92	−0.57	0.64	−0.02	0.02	0.92	−0.43	0.39
Education															
GED, High school graduate	0.05	0.05	0.34	−0.05	0.14	0.02	0.09	0.79	−0.16	0.21	0.07	0.06	0.24	−0.05	0.19
Some college, associate's, vocational/technical school	−0.01	0.04	0.75	−0.10	0.07	−0.07	0.09	0.43	−0.25	0.10	0.00	0.06	1.00	−0.11	0.11
Higher education	−0.09	0.06	0.12	−0.20	0.02	−0.17	0.11	0.13	−0.39	0.52	0.07	0.07	0.34	−0.21	0.07
Minoritizedy ethnic or racial identity	−0.05	0.04	0.17	−0.12	0.02	−0.11	0.07	0.11	−0.25	0.03	0.03	0.05	0.57	−0.12	0.06
Living with disability	0.03	0.04	0.38	−0.04	0.10	0.05	0.07	0.52	−0.09	0.18	0.06	0.05	0.20	−0.03	0.15
Employment															
Parttime and seasonally employed	0.03	0.04	0.45	−0.05	0.10	0.02	0.06	0.72	−0.10	0.15	0.04	0.04	0.31	−0.04	0.13
Employed fulltime	−0.05	0.03	0.12	−0.12	0.01	−0.12	0.06	0.04	−0.23	−0.001	0.04	0.04	0.27	−0.12	0.03
English reading proficiency	0.03	0.02	0.27	−0.02	0.08	0.05	0.05	0.34	−0.49	0.14	0.02	0.03	0.55	−0.04	0.08
Baseline score	0.06	0.01	0.00	0.03	0.09	0.12	0.25	0.00	0.07	0.17	0.06	0.01	0.00	0.03	0.08
Received Housing‐focused DV Services	−0.08	0.03	0.01	−0.15	−0.02	−0.17	0.07	0.01	−0.30	−0.04	0.07	0.04	0.09	−0.16	0.01
Number of children	0.00	0.01	0.73	−0.02	0.03	0.02	0.02	0.41	−0.03	−0.06	0.01	0.01	0.58	−0.04	0.02
Social support	−0.05	0.01	0.00	−0.07	−0.02	−0.10	0.02	0.00	−0.14	−0.06	0.04	0.01	0.00	−0.07	−0.01
Household income	0.00	0.01	0.43	−0.01	0.02	0.05	0.19	0.01	0.11	0.83	0.01	0.01	0.16	0.00	0.02

	Economic abuse					Stalking				
	*b*	*SE*	*p*	95% CI		*b*	*SE*	*p*	95% CI	
Time										
19–24+ months before COVID	0.35	0.07	0.00	0.22	0.49	0.66	0.11	0.00	0.45	0.88
13–18 months before COVID	0.30	0.06	0.00	0.19	0.42	0.55	0.10	0.00	0.36	0.74
7–12 months before COVID	0.22	0.06	0.00	0.11	0.33	0.39	0.09	0.00	0.21	0.57
1–6 months before COVID	0.12	0.06	0.05	0.00	0.23	0.20	0.10	0.03	0.02	0.39
7–12+ months after COVID	0.19	0.27	0.48	−0.34	0.72	0.09	0.46	0.84	−0.80	0.99
Education										
GED, high school graduate	0.04	0.08	0.61	−0.12	0.20	0.03	0.13	0.82	−0.23	0.29
Some college, associate's, vocational/technical school	0.05	0.08	0.51	−0.10	0.20	−0.13	0.12	0.31	−0.37	0.12
Higher education	0.05	0.10	0.58	−0.14	0.25	−0.18	0.16	0.25	−0.49	0.13
Race/ethnicity										
Minority ethnic or racial identity	−0.12	0.06	0.05	−0.24	0.00	−0.10	0.10	0.30	−0.30	0.09
Disability status										
Living with disability	0.05	0.06	0.40	−0.07	0.17	−0.18	0.10	0.06	−0.37	0.01
Employment										
Parttime and seasonally employed	−0.06	0.06	0.33	−0.17	0.06	−0.01	0.09	0.89	−0.20	0.17
Employed fulltime	−0.10	0.05	0.07	−0.20	0.01	−0.10	0.09	0.26	−0.27	0.07
English reading proficiency	0.01	0.04	0.78	−0.07	0.09	−0.03	0.07	0.62	−0.17	0.10
Baseline score	0.13	0.03	0.00	0.07	0.18	0.16	0.03	0.00	0.10	0.21
Housing‐focused DV Services										
Received	−0.16	0.06	0.00	−0.28	−0.05	−0.09	0.09	0.34	−0.27	0.09
Number of children	0.02	0.02	0.37	−0.02	0.06	−0.01	0.03	0.84	−0.07	0.06
Social support	−0.10	0.02	0.00	−0.13	−0.06	−0.11	0.03	0.00	−0.18	−0.05
Household income	0.00	0.01	0.90	−0.02	0.02	−0.02	0.02	0.15	−0.05	0.01

*Note*: Reference Groups: Time—COVID‐19 pandemic onset (0–6 months), Education—less than high school degree, Race/ethnicity —White, Disability status—not living with disability, Employment—unemployed, Housing‐focused DV services—Did not receive.

Abbreviations: CI, confidence interval; DV, domestic violence.

The conditional mixed effect models indicated that three of the time‐invariant and time‐varying covariates significantly predicted all forms of abuse: (1) baseline abuse scores, (2) receipt of the housing‐focused DV services, and (3) social support. Baseline abuse scores were associated with higher levels of abuse, while the housing‐focused DV services and social support were associated with decreased levels of abuse.

### 3.2 Housing instability

**Figure 2:**
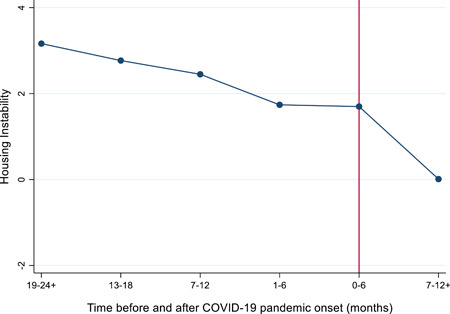
Change in housing instability over time: before and after the onset of COVID‐19. Red line indicates the onset of COVID‐19

The unconditional mixed effect model indicated that housing instability decreased over time before the onset of the COVID‐19 pandemic (Table S3). After the onset of COVID‐19, there were no significant differences between housing instability reported during the first 6 months of the pandemic and the subsequent timepoints (Table 3 and Figure 2).

**Table 3:** Conditional mixed effects models with covariates predicting housing instability

	Housing instability				
	*b*	*SE*	*p*	95% CI	
Time					
19–24+ months before COVID	1.46	0.20	0.00	1.08	1.85
13–18 months before COVID	1.07	0.18	0.00	0.72	1.42
7–12 months before COVID	0.75	0.17	0.00	0.42	1.08
1–6 months before COVID	0.04	0.17	0.82	−0.30	0.38
7–12+ months after COVID	−1.69	0.74	0.02	−3.13	−0.24
Education					
GED, high school graduate	0.34	0.22	0.13	−0.10	0.77
Some college, associate's, vocational/technical school	−0.12	0.21	0.57	−0.53	0.29
Higher education	−0.30	0.27	0.26	−0.82	0.22
Race/ethnicity					
Minority ethnic or racial identity	−0.04	0.17	0.80	−0.37	0.29
Disability status					
Living with disability	−0.01	0.17	0.94	−0.34	0.31
Employment					
Parttime and seasonally employed	−0.03	0.16	0.86	−0.35	0.29
Employed fulltime	−0.33	0.15	0.03	−0.63	−0.03
English reading proficiency	0.16	0.12	0.17	−0.07	0.39
Baseline score	0.22	0.05	0.00	0.13	0.32
Housing‐focused DV Services					
Received	−0.57	0.16	0.00	−0.88	−0.26
Number of children	−0.04	0.05	0.41	−0.15	0.06
Social support	−0.28	0.06	0.00	−0.39	−0.17
Household income	−0.11	0.03	0.00	−0.17	−0.06

*Note*: Reference Groups: Time—COVID‐19 pandemic onset (0–6 months), Education—less than high school degree, Race/ethnicity—White, Disability status—not living with disability, Employment—unemployed, Housing‐focused DV services—did not receive.

Abbreviations: CI, confidence interval; DV, domestic violence.

The conditional mixed effect model indicated that four of the time‐invariant and time‐varying covariates significantly predicted lower housing instability: (1) full‐time employment, (2) receipt of the housing‐focused DV services, (3) higher household income, and (4) social support. Higher baseline scores on housing instability were associated with higher levels of housing instability. These findings suggest that employment, housing‐focused DV services, income, and social support were protective factors against housing instability and homelessness both before and after the onset of COVID‐19.

Interestingly, the conditional mixed effect models also indicated there was a significant difference in housing instability during the initial 6 months of the pandemic and 7–12+ months after the onset of pandemic (Table 3 and Figure 2). Specifically, housing instability later in the pandemic was significantly lower, suggesting a return to the prior downward trajectory.

### 3.3 Mental health

**Figure 3:**
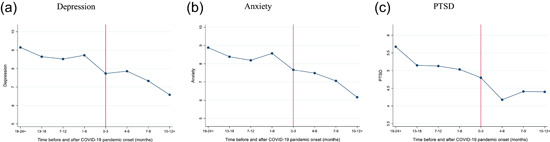
(a–c) Change in mental health over time: before and after the onset of COVID‐19. Red line indicates the onset of COVID‐19

Results from the unconditional mixed effect models indicated that depression and anxiety levels were decreasing before the onset of the COVID‐19 (Table S4). Regarding the effects of the COVID‐19 pandemic, results suggest there were no significant differences between depression and anxiety levels reported during the first 3 months of pandemic onset and during the subsequent timepoints, suggesting that depression and anxiety levels may have stabilized during the pandemic (Table 4 and Figure 3a,b).

**Table 4:** Conditional mixed effects models with covariates predicting mental health

	Depression					Anxiety					PTSD				
	*b*	*SE*	*p*	95% CI		*b*	*SE*	*p*	95% CI		*b*	*SE*	*p*	95% CI	
Time															
19–24+ months before COVID	1.41	0.68	0.04	0.07	2.76	1.22	0.64	0.06	−0.03	2.47	0.88	0.34	0.01	0.20	1.55
13–18 months before COVID	0.91	0.56	0.10	−0.18	2.01	0.72	0.52	0.17	−0.30	1.74	0.35	0.28	0.21	−0.20	0.90
7–12 months before COVID	0.79	0.50	0.12	−0.20	1.77	0.52	0.47	0.26	−0.39	1.44	0.33	0.25	0.19	−0.16	0.82
1–6 months before COVID	0.99	0.47	0.04	0.06	1.92	0.90	0.44	0.04	0.04	1.77	0.24	0.24	0.32	−0.23	0.70
4–6 months after COVID	0.13	0.73	0.86	−1.31	1.56	−0.18	0.69	0.79	−1.53	1.17	−0.62	0.37	0.09	−1.34	0.10
7–9 months after COVID	−0.40	0.70	0.56	−1.77	0.96	−0.61	0.65	0.35	−1.88	0.67	−0.39	0.35	0.27	−1.07	0.30
10–12+ months after COVID	−1.15	0.84	0.17	−2.79	0.49	−1.50	0.80	0.06	−3.06	0.06	−0.40	0.43	0.35	−1.24	0.44
Education															
GED, high school graduate	0.00	0.67	1.00	−1.31	1.31	−0.25	0.63	0.69	−1.49	0.99	−0.16	0.35	0.64	−0.84	0.52
Some college, associate's, vocational/technical school	0.92	0.63	0.14	−0.32	2.15	0.44	0.60	0.46	−0.73	1.62	0.34	0.33	0.30	−0.31	0.99
Higher education	−0.53	0.84	0.53	−2.18	1.13	0.02	0.80	0.98	−1.55	1.59	−0.32	0.44	0.47	−1.19	0.55
Race/ethnicity															
Minority ethnic or racial identity	0.56	0.52	0.29	−0.47	1.58	0.18	0.50	0.73	−0.81	1.16	−0.14	0.28	0.61	−0.68	0.40
Disability status															
Living with disability	1.63	0.53	0.00	0.60	2.66	1.58	0.50	0.00	0.60	2.56	0.45	0.28	0.10	−0.09	0.99
Employment															
Parttime and seasonally employed	−0.83	0.49	0.09	−1.79	0.14	0.00	0.56	0.99	−0.90	0.90	0.18	0.25	0.48	−0.31	0.66
Employed fulltime	−1.12	0.46	0.02	−2.03	−0.21	−1.00	0.43	0.02	−1.85	−0.15	−0.28	0.23	0.24	−0.73	0.18
English reading proficiency	0.27	0.34	0.43	−0.40	0.95	0.36	0.33	0.27	−0.28	1.01	−0.02	0.18	0.92	−0.38	0.34
Baseline score	0.35	0.04	0.00	0.28	0.43	0.41	0.36	0.00	0.34	0.48	0.58	0.05	0.00	0.49	0.68
Housing‐focused DV Services															
Received	−1.17	0.48	0.02	−2.12	−0.23	−0.57	0.46	0.22	−1.47	0.33	−0.46	0.26	0.07	−0.96	0.04
Number of children	−0.01	0.05	0.83	−0.11	0.08	0.08	0.05	0.10	−0.01	0.17	0.05	0.02	0.06	0.00	0.09
Social support	−1.16	0.17	0.00	−1.50	−0.83	−1.28	0.16	0.00	−1.59	−0.96	−0.44	0.09	0.00	−0.61	−0.26
Household income	−0.02	0.09	0.78	−0.20	0.15	0.02	0.08	0.79	−0.14	0.18	0.00	0.05	0.95	−0.09	0.09

*Note*: Reference Groups: Time—COVID‐19 pandemic onset (0–3 months), Education—less than high school degree, Race/ethnicity—White, Disability status—not living with disability, Employment—unemployed, Housing‐focused DV Services—did not receive.

Abbreviations: CI, confidence interval; DV, domestic violence.

The conditional mixed effect models indicated that several of the time‐invariant and time‐varying covariates significantly predicted depression and anxiety levels (Table 4). Higher baseline scores and living with a disability were associated with higher levels of depression and anxiety. Additionally, being employed full‐time, receiving housing‐focused DV services, and having access to social support were associated with lower levels of depression and anxiety. Interestingly, after accounting for the covariates, there were no longer significant differences between depression and anxiety reported at pandemic onset, 7–12 and 13–18 months before the onset of the COVID‐19 pandemic. These findings suggest that employment, housing‐focused DV services, and social support were protective factors.

For PTSD symptomology, the conditional mixed effects models evidenced a similar trajectory—PTSD symptoms during the 13–18 and 19–24+ months before the COVID‐19 pandemic were significantly higher than at the pandemic onset (Figure 3c). There were no significant differences between PTSD symptoms during the onset of the pandemic and 4–6 months, 7–9 months, or 10–12+ months after the onset of the pandemic. Additionally, conditional mixed effect models indicated that social support were significantly associated with lower levels of PTSD and higher baseline housing instability scores were associated with higher levels of PTSD.

## 4 DISCUSSION

The purpose of this analysis was to explore the impact that the COVID‐19 pandemic had on the safety, housing stability, and mental health of a racially diverse sample of unstably housed DV survivors over time. Our study was uniquely positioned to address this research question due to the timing of the interviews spaced 6 months apart. Some participants went through the entirety of the 24‐month study before the onset of COVID‐19, others experienced it early in the study, and others towards the end of the study. At baseline, all participants had recently experienced DV, were in unstable housing conditions, and had recently reached out to a DV agency for support. Our findings suggest that participants were improving before the pandemic onset. After the onset of the COVID‐19 pandemic, improvements on safety, housing stability, and mental health all plateaued.

A critical finding of this study is that experiences of abuse, housing instability, and mental health symptomatology did not worsen as a result of the COVID‐19 stay‐at‐home orders. This finding runs contrary to much of the empirical research released early in the pandemic. Numerous studies throughout 2020 and early 2021 (Leslie & Wilson, 2020; Marroquín et al., 2020; Nix & Richards, 2021; Piquero et al., 2020; Sharma & Borash, 2020) and research examining DV during other large‐scale crises (e.g., Rauhaus, et al., 2020) showed an increase in DV during pandemics and other global catastrophes, as well as increases in mental health symptomatology (Almeida et al., 2020; Bhattacharjee & Acharya, 2020). The most likely explanation for our results may be found in the makeup of our sample: DV survivors who were experiencing homelessness or housing instability at the onset of the study, but who had made connections with DV agencies. These individuals had sought help to stay safe, and the majority were no longer living with their abusive partner by the time COVID‐19 started. Studies examining the rise in DV as a result of the pandemic were generally focused on survivors who were living with, and, therefore, quarantined with, their abusive partners.

Although victimization, housing instability and mental health symptomatology did not worsen for this population as a result of COVID‐19, progress that was being made in each of these areas plateaued. This is likely directly related to the need for social distancing to stop the spread of COVID‐19. Governmental orders and healthcare recommendations for social distancing, for example, resulted in DV agencies suspending some or all their in‐person services, at least temporarily, and for a period of time people were asked to not even leave their homes. For those living with their abusive partner when COVID‐19 began, they had no way out and little if any support if abuse continued. For those who had no contact with and who were not being abused by the abusive partner when stay‐at‐home orders were implemented, there was less likelihood of abuse escalating over time.

The pandemic also stalled movement in housing stability. With the implementation of precautionary shutdown measures, employee layoffs, and furloughs, many states executed emergency housing payment assistance, temporary eviction freezes, and moratoriums on rent increases and foreclosure as direct interventions to mitigate the effects of the economic disruptions caused by the pandemic (Layser et al., 2020). These temporary measures likely prevented significant increases in housing instability, especially for study participants enrolled in affordable government rental housing programs before the pandemic. However, the precautionary shutdown of DV agencies, particularly those providing housing services, likely prevented significant decreases in housing instability as study participants in dire need of housing advocacy may have been unable to access services to improve their living conditions (Su et al., 2021). Similarly, the return to the downward trajectory of housing instability observed in this study after the first 6‐month post‐COVID‐19 timeframe aligns with the resumption of DV agency housing services and the availability of additional governmental COVID‐19 housing and homelessness services including rental assistance to further mitigate the economic impacts of the pandemic.

Consistent with the findings regarding safety and housing stability, survivors' mental health plateaued as a result of COVID‐19. While depression, anxiety and PTSD were all going down over time before the pandemic, positive change stalled in its aftermath. While it was somewhat surprising that depression, anxiety, and PTSD did not increase as a result of COVID‐19, as found in other studies (e.g., Bhattacharjee & Acharya, 2020; Sediri et al., 2020), the fact that they stopped decreasing can be viewed as a negative outcome. Mental health was also impacted by several other factors worthy of discussion. For example, living with a physical disability or disabling condition was significantly associated with higher mental health symptomatology and stalking victimization. This finding is in line with previous research showing adults with disabilities report higher rates of mental distress than those without disabilities, wherein more than 1/3 of adults with disabilities report experiencing frequent mental distress (Cree et al., 2020). These higher rates of mental distress are likely associated with the higher rates of DV also observed among the disabled community which includes sexual and physical violence, psychological aggression, stalking and control of reproductive health (Breiding & Armour, 2015).

Finally, participants who had more social support reported less abuse, less housing instability, and lower mental health distress. Prior research has clearly demonstrated the protective nature of social support on DV victimization (Beeble et al., 2009; Dias et al., 2019). Within the context of the COVID‐19 pandemic, mandatory lockdowns may have isolated survivors by restricting their access to social support (Wood et al., 2021). Further, evidence suggests that abusive partners may have used the mandatory lockdowns to limit survivors' access to social support (National Domestic Violence Hotline, 2020). Taken together, our findings build on prior research emphasizing the importance of informal and formal social supports for DV survivors navigating transitions to safety, but importantly, in this study social support was especially impactful in mitigating the devastating social impact of COVID‐19.

## 5 LIMITATIONS

Several limitations must be considered when interpreting these findings. First and foremost, our sample was entirely composed of homeless or unstably housed individuals who had sought help from a DV support agency at the time of study entry. Indeed, the majority of our sample received housing‐focused DV services in the first 6 months of the study that resulted in numerous positive life changes. Further, the majority of participants were no longer living with the person who had abused them at the time COVID‐19 began. Therefore, our sample is not representative of all DV survivors. Further, the recall periods and interview schedule limited our ability to examine month by month changes in our outcomes. As such, we were unable to capture changes reflective of discrete COVID‐related events (e.g., receipt of stimulus checks) that may have had significant effects on the outcomes assessed. Finally, this study did not include a comparison group of DV survivors who had not sought services. Therefore, all positive changes that we propose are due to their receipt of services may, in fact, be due at least in part to the passage of time.

## 6 POLICY AND PRACTICE IMPLICATIONS

These findings have important implications for policy and practice. As the threat of future pandemics is very real, it is critical to understand how such pandemics impact various populations—especially those who are multiply marginalized and oppressed. This study provides a number of lessons for moving forward.

First, this study provides evidence that DV services may mitigate the negative outcomes associated with the pandemic. The study participants had been experiencing numerous positive life changes before the onset of COVID‐19, and while progress was temporarily stalled, people's lives did not substantially worsen either. Further, once the first 6 months of the pandemic had concluded, the positive trajectory of housing stability resumed.

While the receipt of any service from the DV agencies appears to have been helpful, access specifically to housing‐focused DV services (housing‐focused advocacy and/or direct funding assistance) was uniquely influential in reducing all forms of abuse except stalking and was related to greater housing stability and lower mental health symptomatology. This provides further evidence for the effectiveness of the DV Housing First model, which involves the use of housing‐focused advocacy and financial assistance to increase the safety and housing stability of DV survivors (Sullivan & Olsen, 2016; Thomas et al., 2020). More funding is needed to go to agencies so that they can provide individualized financial resources to survivors and so they have enough advocates to provide time‐intensive housing advocacy (Sullivan et al., 2019). If such efforts lead to increased safety, housing stability and well‐being for survivors, such expenditures will be extremely cost effective.

The pandemic forced many human service agencies, including DV agencies, to do their work differently. Many increased their use of technology so they could continue providing services remotely (Emezue, 2020) and, as such, can reach survivors who may otherwise find it problematic or difficult to engage in services. Agency staff can still provide emotional support, information, referrals and even advocacy through the telephone, social media platforms, and video conferencing. Given the importance of services in decreasing abuse, increasing housing stability and decreasing mental health symptomatology, it is critical that DV agencies continue engaging in practices that will reach more diverse and multiply marginalized survivors.

The role of social support in enhancing survivors' safety, housing stability and mental health was also a critical finding with practice implications. This finding aligns with a wealth of prior evidence linking social support with positive outcomes for survivors (e.g., Beeble et al., 2009; Goodman & Smyth, 2011; Jose & Novaco, 2016; Kamimura et al., 2013), and suggests that DV agencies should prioritize this focus with survivors regardless of whether a pandemic is occurring. Goodman et al. (2016) have identified, through focus groups with advocates, the importance of helping survivors to reconnect to existing networks and build new helpful networks. Such efforts can be done in‐person as well as remotely, and do not require additional resources other than knowledge and skills. Our findings further emphasize the importance of both formal and informal support networks for DV survivors, particularly in the face of global catastrophes.

The moratorium on evictions, as well as the stimulus checks, likely also worked to mitigate the negative consequences of the pandemic. While this study was unable to directly test these relationships, the fact that housing instability did not worsen during the pandemic suggests these policies were effective in reducing homelessness.

## 7 RESEARCH IMPLICATIONS

Examining the role of a global pandemic on DV survivors' well‐being is critical not just because such a pandemic or other large‐scale crisis is likely to reoccur, but because there are lessons to be learned from the mechanisms through which the pandemic impacted individuals, families, and communities. The social distancing mandates, for example, while effective in reducing the spread of a deadly virus, led to greater isolation and fewer community‐based resources and support. The experience of the pandemic was not felt equally by all, with some people staying relatively unscathed (those with high incomes and jobs they could work remotely, and who had resources to shield themselves from most of the negative consequences). Those most negatively impacted by the pandemic were those who already had fewer resources, supports and protections at the start (Rauhaus et al. 2020). As community psychologists, it is critical that we closely consider the multiple intersecting relationships, systems, and contexts that affect people's experience of DV as well as other forms of violence or hardships. Moving forward, researchers should be prepared for unexpected events and contexts (e.g., pandemics, natural disasters, change in political structures) that can impact people's experiences and well‐being. In this study, researching the pandemic's impact through a social justice lens was one such opportunity to conduct a study that may lead to positive social change.

## 8 CONCLUSIONS

The purpose of this study was to examine the impact of COVID‐19 on a racially and ethnically diverse sample of homeless and unstably housed DV survivors. Although we expected the pandemic to contribute to increased DV victimization, housing instability, and mental health symptomatology, our results suggest that participants did not get worse, but rather their progress toward achieving safety and stability was briefly stalled during the first year of the pandemic. These findings provide critical insight into the importance of service access during and after global catastrophes. They also highlight the importance of accounting for the interplay of multiple factors that influence the severity of the pandemic on the lives of DV survivors.

## CONFLICT OF INTERESTS

The authors declare that there are no conflict of interests.

### PEER REVIEW

The peer review history for this article is available at https://publons.com/publon/10.1002/jcop.22765

## Supporting information

Supporting information.

## Data Availability

The data that support the findings of this study are available on request from the corresponding author. The data are not publicly available due to privacy or ethical restrictions.
