# Supplementary material for: The impact of COVID‐19 on the safety, housing stability, and mental health of unstably housed domestic violence survivors
Source: J Community Psychol. 2021 Dec 18;50(6):2659–81. doi: 10.1002/jcop.22765 (PMC9206039; doi:10.1002/jcop.22765)
Supplement: Supplementary file 1 — Supporting information. [file JCOP-50-2659-s001.docx]

Table S1. Data Restructuring

| Timepoint | Original Data Structure |  | Restructured Dataset 1 |  | Restructured Dataset 2 |
| --- | --- | --- | --- | --- | --- |
| 1 | Baseline Interview |  | 19-24+ months before COVID |  | 19-24+ months before COVID |
| 2 | 6 months after baseline |  | 13-18 months before COVID |  | 13-18 months before COVID |
| 3 | 12 months after baseline |  | 7-12 months before COVID |  | 7-12 months before COVID |
| 4 | 18 months after baseline |  | 1-6 months before COVID |  | 1-6 months before COVID |
| 5 | 24 months after baseline |  | 0-6 months after COVID |  | 0-3 months after COVID |
| 6 | -- |  | 7-12+ months after COVID |  | 4-6 months after COVID |
| 7 | -- |  | -- |  | 7-9 months after COVID |
| 8 | -- |  | -- |  | 10-12+ months after COVID |

Table S2. Unconditional mixed effects models predicting abuse

|  | Physical Abuse | | | | |  | Emotional Abuse | | | | |  | Sexual Abuse | | | | | | | |
| --- | --- | --- | --- | --- | --- | --- | --- | --- | --- | --- | --- | --- | --- | --- | --- | --- | --- | --- | --- | --- |
|  | b | SE | p | 95% CI | |  | b | SE | p | 95% CI | |  | b | | SE | | p | | 95% CI | |
| Time |  |  |  |  |  |  |  |  |  |  |  |  |  |  | |  | |  | |  |
| 19-24+ months before COVID | 0.13 | 0.05 | 0.00 | 0.04 | 0.22 |  | 0.25 | 0.07 | 0.00 | 0.11 | 0.39 |  | 0.11 | 0.05 | | 0.03 | | 0.01 | | 0.21 |
| 13-18 months before COVID | 0.10 | 0.04 | 0.02 | 0.02 | 0.18 |  | 0.21 | 0.06 | 0.00 | 0.08 | 0.33 |  | 0.08 | 0.08 | | 0.07 | | -0.01 | | 0.17 |
| 7-12 months before COVID | 0.04 | 0.04 | 0.31 | -0.04 | 0.11 |  | 0.10 | 0.06 | 0.08 | -0.01 | 0.22 |  | 0.10 | 0.10 | | 0.01 | | 0.02 | | 0.19 |
| 1-6 months before COVID | 0.00 | 0.04 | 0.94 | -0.08 | 0.07 |  | 0.04 | 0.06 | 0.56 | -0.08 | 0.15 |  | 0.02 | 0.02 | | 0.64 | | -0.06 | | 0.11 |
| 7-12+ months after COVID | -0.05 | 0.17 | 0.77 | -0.38 | 0.28 |  | 0.04 | 0.26 | 0.89 | -0.47 | 0.54 |  | 0.02 | 0.02 | | 0.93 | | -0.34 | | 0.38 |

*Note.* COVID-19 pandemic onset (0-6 months after COVID) used as the reference group.

Table S2. Unconditional mixed effects models predicting abuse (continued)

|  | Economic Abuse | | | | |  | Stalking | | | | |
| --- | --- | --- | --- | --- | --- | --- | --- | --- | --- | --- | --- |
|  | b | SE | p | 95% CI | |  | b | SE | p | 95% CI | |
| Time |  |  |  |  |  |  |  |  |  |  |  |
| 19-24+ months before COVID | 0.40 | 0.07 | 0.00 | 0.27 | 0.53 |  | 0.66 | 0.11 | 0.00 | 0.46 | 0.87 |
| 13-18 months before COVID | 0.28 | 0.06 | 0.00 | 0.17 | 0.40 |  | 0.52 | 0.09 | 0.00 | 0.33 | 0.71 |
| 7-12 months before COVID | 0.21 | 0.05 | 0.00 | 0.11 | 0.32 |  | 0.32 | 0.09 | 0.00 | 0.14 | 0.49 |
| 1-6 months before COVID | 0.12 | 0.06 | 0.03 | 0.01 | 0.23 |  | 0.15 | 0.39 | 0.09 | -0.03 | 0.33 |
| 7-12+ months after COVID | 0.15 | 0.23 | 0.51 | -0.30 | 0.61 |  | 0.09 | 0.09 | 0.82 | -0.67 | 0.85 |

*Note.* COVID-19 pandemic onset (0-6 months) used as the reference group.

Table S3. Unconditional mixed effects models predicting housing instability

|  | Housing Instability | | | | |  |
| --- | --- | --- | --- | --- | --- | --- |
|  | b | SE | p | 95% CI | | |
| Time |  |  |  |  |  | |
| 19-32+ months before COVID | 1.60 | 0.19 | 0.00 | 1.22 | 1.98 | |
| 13-18 months before COVID | 1.13 | 0.17 | 0.00 | 0.79 | 1.47 | |
| 7-12 months before COVID | 0.73 | 0.16 | 0.00 | 0.41 | 1.05 | |
| 1-6 months before COVID | 0.11 | 0.17 | 0.51 | -0.22 | 0.44 | |
| 7-12+ months after COVID | -1.04 | 0.65 | 0.11 | -2.32 | 0.24 | |

*Note.* COVID-19 pandemic onset (0-6 months) used as the reference group.

Table S4. Unconditional mixed effects models predicting mental health

|  | Depression | | | | |  | Anxiety | | | | |  | | | PTSD | | | | |
| --- | --- | --- | --- | --- | --- | --- | --- | --- | --- | --- | --- | --- | --- | --- | --- | --- | --- | --- | --- |
|  | b | SE | p | 95% CI | |  | b | SE | p | 95% CI | | |  | | b | SE | p | 95% CI | |
| Time |  |  |  |  |  |  |  |  |  |  |  | | |  |  |  |  |  |  |
| 19-24+ months before COVID | 1.67 | 0.61 | 0.01 | 0.46 | 2.88 |  | 1.74 | 0.57 | 0.00 | 0.63 | 2.85 | | |  | 1.37 | 0.29 | 0.00 | 0.81 | 1.94 |
| 13-18 months before COVID | 1.54 | 0.50 | 0.00 | 0.57 | 2.52 |  | 1.18 | 0.46 | 0.01 | 0.29 | 2.09 | | |  | 0.46 | 0.23 | 0.05 | 0.01 | 0.92 |
| 7-12 months before COVID | 0.94 | 0.45 | 0.04 | 0.06 | 1.82 |  | 0.78 | 0.42 | 0.06 | -0.03 | 1.60 | | |  | 0.32 | 0.21 | 0.12 | -0.09 | 0.74 |
| 1-6 months before COVID | 1.13 | 0.42 | 0.01 | 0.31 | 1.95 |  | 1.00 | 0.38 | 0.01 | 0.24 | 1.75 | | |  | 0.19 | 0.19 | 0.33 | -0.19 | 0.57 |
| 4-6 months after COVID | 0.64 | 0.69 | 0.35 | -0.71 | 1.99 |  | 0.52 | 0.64 | 0.41 | -0.74 | 1.77 | | |  | -0.36 | 0.32 | 0.26 | -1.00 | 0.27 |
| 7-9 months after COVID | -0.28 | 0.59 | 0.64 | -1.45 | 0.88 |  | -0.73 | 0.55 | 0.90 | -1.15 | 1.00 | | |  | -0.47 | 0.27 | 0.09 | -1.02 | 0.08 |
| 10+ months after COVID | -0.30 | 0.76 | 0.69 | -1.80 | 1.19 |  | -0.54 | 0.71 | 0.45 | -1.94 | 0.85 | | |  | -0.32 | 0.37 | 0.38 | -1.04 | 0.40 |

*Note.* COVID-19 pandemic onset (0-3 months) used as the reference group.
